# Protective effect of APOE epsilon 2 on intrinsic functional connectivity of the entorhinal cortex is associated with better episodic memory in elderly individuals with risk factors for Alzheimer's disease

**DOI:** 10.18632/oncotarget.11289

**Published:** 2016-08-14

**Authors:** Jiu Chen, Hao Shu, Zan Wang, Duan Liu, Yongmei Shi, Lin Xu, Zhijun Zhang

**Affiliations:** ^1^ Department of Neurology, Affiliated ZhongDa Hospital, Medical School, Southeast University, Nanjing, Jiangsu, China; ^2^ Key Laboratory of Animal Models and Human Disease Mechanisms, Chinese Academy of Sciences, Kunming Institute of Zoology, Kunming, Yunnan, China; ^3^ Department of Psychology, Xinxiang Medical University, Xinxiang, Henan, China

**Keywords:** amnestic mild cognitive impairment, apolipoprotein E, entorhinal cortex, fMRI, functional connectivity, Gerotarget

## Abstract

The apolipoprotein E (APOE) ε4 allele associates with accelerating the conversion from amnestic mild cognitive impairment (aMCI) to Alzheimer's disease (AD), whereas the protectiveAPOEε2 allele appears to be against the disease. Moreover, entorhinal cortex (ERC) is one of the earliest brain regions of AD pathology that disrupts the formation of episodic memory. To investigate the effects of APOE ε2 and ε4alleles on functional connectivity (FC) of ERC and cognition in aMCI. Methods The FC analyses of ERC were performed in 83 aMCI (9 ε2-carrier, 44 ε3ε3, and 30 ε4-carrier) and 88 healthy controls (HC, 15 ε2-carrier, 40 ε3ε3, and 33 ε4-carrier). Multiple linear regression model was performed between the altered ERC connectivities and cognition. In the ERC network, aMCI with ε4-carriers showed decreased FC in the bilateral middle temporal gyrus (MTG), right precuneus, and right precentral gyrus (PreCG), while ε2-carriers showed increased FC in these regions (except the right PreCG) compared to HC. The altered FC between ERC and right MTG correlated with episodic memory performance in aMCI carried ε2 and ε4 alleles. These results suggest that the effects of*APOE*on the ERC network are closely linked to the role of this gene on AD risk, which aMCI with ε4-carriers can accelerate the pathological progression of network-based mechanisms while ε2-carriers may play a protective role in contributing to a compensatory mechanism. It further suggests that APOE can appear to directly affect the ERC-MTG neural pathway associated with the impairment of episodic memory in aMCI.

## INTRODUCTION

Mild cognitive impairment (MCI), as a high-risk factor for dementia due to Alzheimer's disease (AD), is characterized by the disruption of episodic memory formation and is usually thought to reflect a transitional state between normal aging and dementia due to AD [1, 2], and can be further classified into subtypes including amnestic MCI (aMCI)/non-amnestic MCI (naMCI) and single/multidomain MCI [3, 4]. The aMCI-multiple domain is consistently considered to have a higher conversion rate to AD compared to other MCI subtypes [5, 6]. It is well-known that the entorhinal cortex (ERC) is one of the earliest brain regions of AD pathology [7], in concert with the hippocampus, and plays a pivotal role in the normal formation of episodic memory [8]. Recently, resting-state intrinsic functional connectivity magnetic resonance imaging (rs-fcMRI), measured by spatial synchronization of blood oxygenation level-dependent signal fluctuation, is consistently considered as a particularly useful technique not only for detecting changes in brain function that are present very early in the progression of AD but also in predicting cognitive performance [9-11]. Using rs-fcMRI, changes in the functional connectivity (FC) of several networks have been identified in patients with aMCI and AD [9, 10, 12]. However, it still remains unknown about the role of ERC network for revealing potential network-based disease mechanisms in aMCI.

Converging clinical and pathological evidence has consistently indicated the apolipoprotein E (APOE) ε4 allele is the best established genetic risk factor for the progression of early AD [13-15], accelerating episodic memory decline [16], and conversion of aMCI to AD [17], whereas the protective APOEε2 allele appears to be against the disease [14, 15, 18, 19], and improvement of episodic memory over time [20]. Moreover, our previously published study also suggests that the effects of*APOE*on brain gray matter (GM) volumes in aMCI are closely linked to the role of this gene on AD risk [21]. Recently, rs-fcMRI has also been used to examine the effects of*APOE*on normal brain function [22-24], which suggests that *APOE*genotypes influence the FC of the resting brain. Therefore, converging evidence suggests that APOE polymorphism can affect multiple network-based physiopathologic pathways and is extremely divergent on pathological cognitive change in aMCI [25].

Recently, numerous studies have indicated that the dysfunction and local deformations of ERC can be an early predictor of conversion from aMCI to AD [26, 27]. For example, ERC cortical thinning early in the course of AD is related to the accumulation of neurofibrillary tangles,[28] smaller ERC volume in aMCI is an early predictor of conversion to AD [26], and the impaired ERC is related to the dysfunction of episodic memory [29]. Furthermore, several rs-fcMRI studies have reported an increased FC between ERC and the medial temporal lobe [27]. Previous studies reveal connectivity changes in APOEε4-carriers decades prior to the typical age of onset of clinical symptoms of AD [30]. More importantly, recent functional neuroimaging studies have identified the specific properties and roles of ERC network for revealing potential disease mechanisms [31]. Although some studies have reported that *APOE*genotypes differentially influence the intrinsic FC of the resting brain in aMCI [32], these studies examining the effects of this gene have compared only ε4-carriers with non-carriers. There has been no investigation of the effect of the ε2 allele on intrinsic FC. Furthermore, evidence linking APOE ε4 allele to resting-state FC in aMCI is limited, and previous studies either did not include a network-based brain-behavior relationship or included relatively few participants, limiting the statistical power. More especially, no*APOE*-fMRI research has focused on the ERC network in aMCI, which is closely associated with the episodic memory formation.

**Figure 1:**
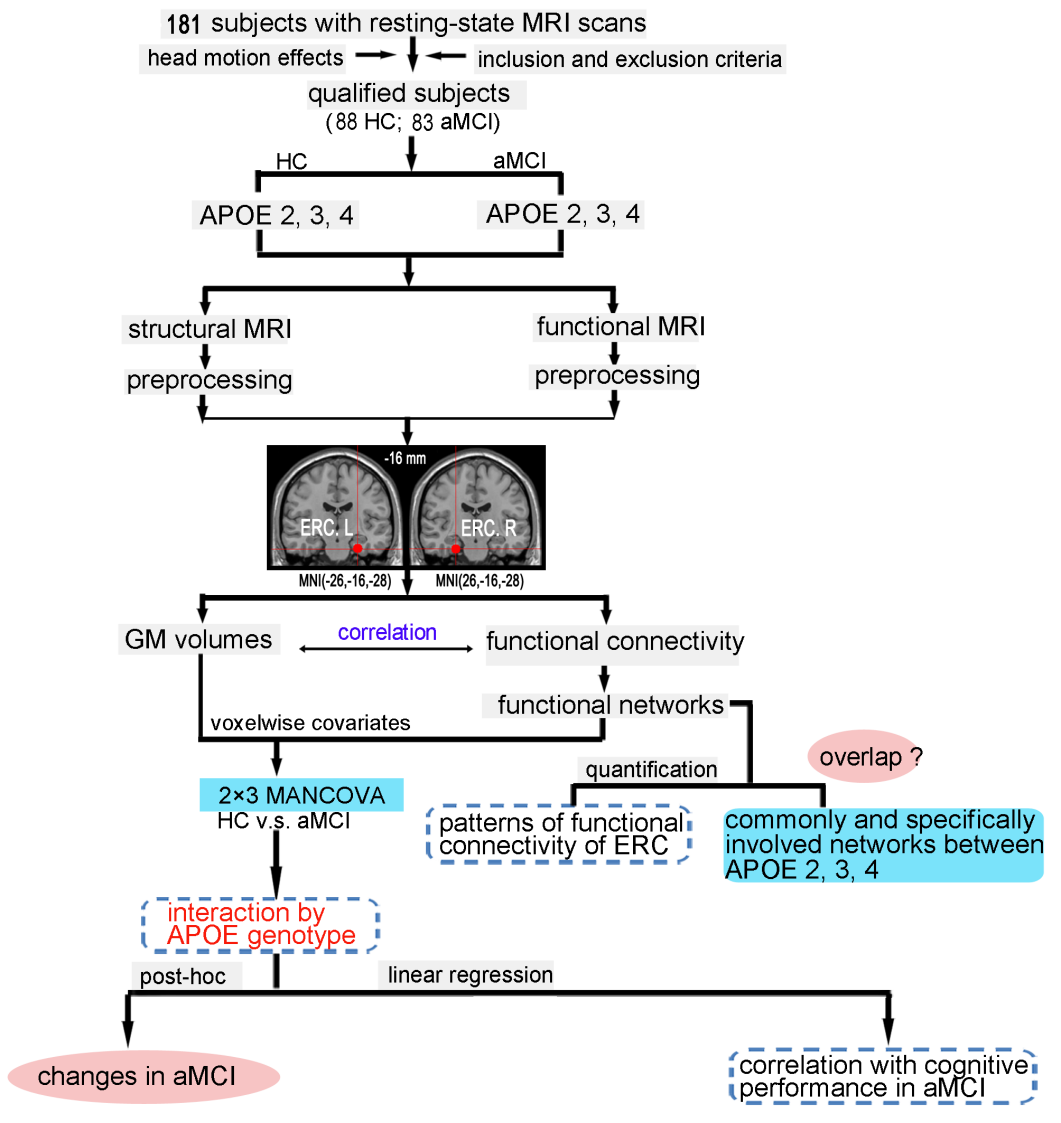
Schematic of data analysis pipeline Regional mean fMRI time series were estimated by a custom T1 template, which was built by averaging the normalized anatomical images across all subjects. The 2×3 MANCOVA analyses were used to compare the differences between aMCI and HC subjects on grey matter volumes and functional connectivity; the multiple linear regression was used to examine the effect of APOE genotype on the association between the functional connectivity and cognitive performance in aMCI subjects. Furthermore, schematic polar plots were used to summarize overall functional connectivity patterns of ERC with target regions throughout the whole-brain. Abbreviations: ERC, entorhinal cortex; fMRI, functional magnetic resonance imaging; HC, healthy controls; aMCI, amnestic mild cognitive impairment; MANCOVA, multivariate analysis of covariance.

The aim of this study was therefore to investigate the effect of the APOE polymorphism with aMCI on intrinsic FC in the ERC network, and to evaluate the effect of the APOEε2 and APOEε4 alleles on the associations between the altered ERC connectivity in aMCI compared to healthy controls (HC) and cognition. We hypothesized that the APOE ε4-carriers would have a decreased FC of the ERC network while the APOE ε2-carriers have the increase with respect to the APOEε3 homozygotes in aMCI compared to HC. And we further predicted that the intrinsic FC pathway underlying differential effects of APOE polymorphism was associated with the impairment of episodic memory in aMCI. As shown in Figure 1, a flowchart summarizes the experimental procedures conducted in this study.

## RESULTS

### Demographic and neuropsychological characteristics

There were no significant differences in age, gender, or education levels between the aMCI and HC (*p* > 0.05). Compared with HC, aMCI exhibited significantly lower MMSE and MDRS scores and significant impairments in episodic memory, information processing speed, executive function, and visuospatial cognition (*p* < 0.05, Bonferroni corrected, Table 1).

**Table 1:** Demographics and clinical measures of patients with aMCI and HC subjects

	HC			aMCl				
	ε2 carrier	ε3/e3	ε4 carrier	ε2 carrier	ε3/e3	ε4 carrier	F values	*p* values
	*n*=15	*n*=40	*n*=33	*n*=9	*n*=44	*n*=30	(χ^2^)	
Age (years)	69.8(6.1)	68.9(5.3)	68.3(6.2)	64.8(7.7)	68.9(7.4)	71.0(6.9)	0.370	0.544
Gender (male/female)	6/9	17/23	16/17	4/5	26/18	15/15	1.897	0.387
Education level (years)	12.6(3.4)	12.8(3.0)	11.8(2.6)	10.9(2.1)	12.1(3.5)	11.5(2.9)	2.553	0.112
MMSE scores	27.7(1.9)	28.4(1.1)	28.1(1.4)	27.4(1.4)	26.3(2.6)	25.8(3.0)	16.250	<0.001*
MDRS-2	138.1(2.9)	137.8(3.7)	137.5(3.3)	136.1(5.7)^c^	131.5(6.8)	129.5(6.9)	15.192	<0.001*
**Composite Z scores of each cognitive domain**								
Episodic memory	0.34(0.4)	0.52(0.5)	0.59(0.5)	-0.32(0.6)	-0.65(0.6)	-0.91(0.7)	39.177	<0.001*
information processing speed	0.04(0.4)	-0.14(0.4)	-0.11(0.3)	-0.03(0.4)	0.24(0.6)	0.20(0.7)	4.007	0.002*
Executive function	0.11(0.3)	0.12(0.4)	0.16(0.5)	-0.30(0.3)	-0.05(0.4)	-0.08(0.4)	12.764	<0.001*
Visuospatial function	0.20(0.7)	0.13(0.6)	0.26(0.6)	0.25(0.6)^c^	-0.05(0.7)	-0.66(1.2)	6.011	<0.001*

Notes: Values are expressed as the mean (standard deviation, SD). Abbreviations: MMSE, Mini mental state exam; MDRS-2, Mattis Dementia Rating Scale-2; HC, healthy controls; aMCI, amnestic mild cognitive impairment.

* Signifcant differences were found between aMCI and HC. P values were obtained by MANCOVA analysis except for gender (chi square test).

*a-c: post-hoc analysis* (LSD test for demographic information and Bonferroni correction for multiple comparison) further revealed the source of MANCOVA difference *(a: APOE2 vs. APOE3; b: APOE3 vs. APOE4; c: APOE2 vs. APOE4) APOE2, ε2 carrier; APOE3, ε3/ε3; APOE4, ε4 carrier.*

### Disrupted functional connectivity of ERC network on the interaction of APOE with aMCI patients

**Figure 2:**
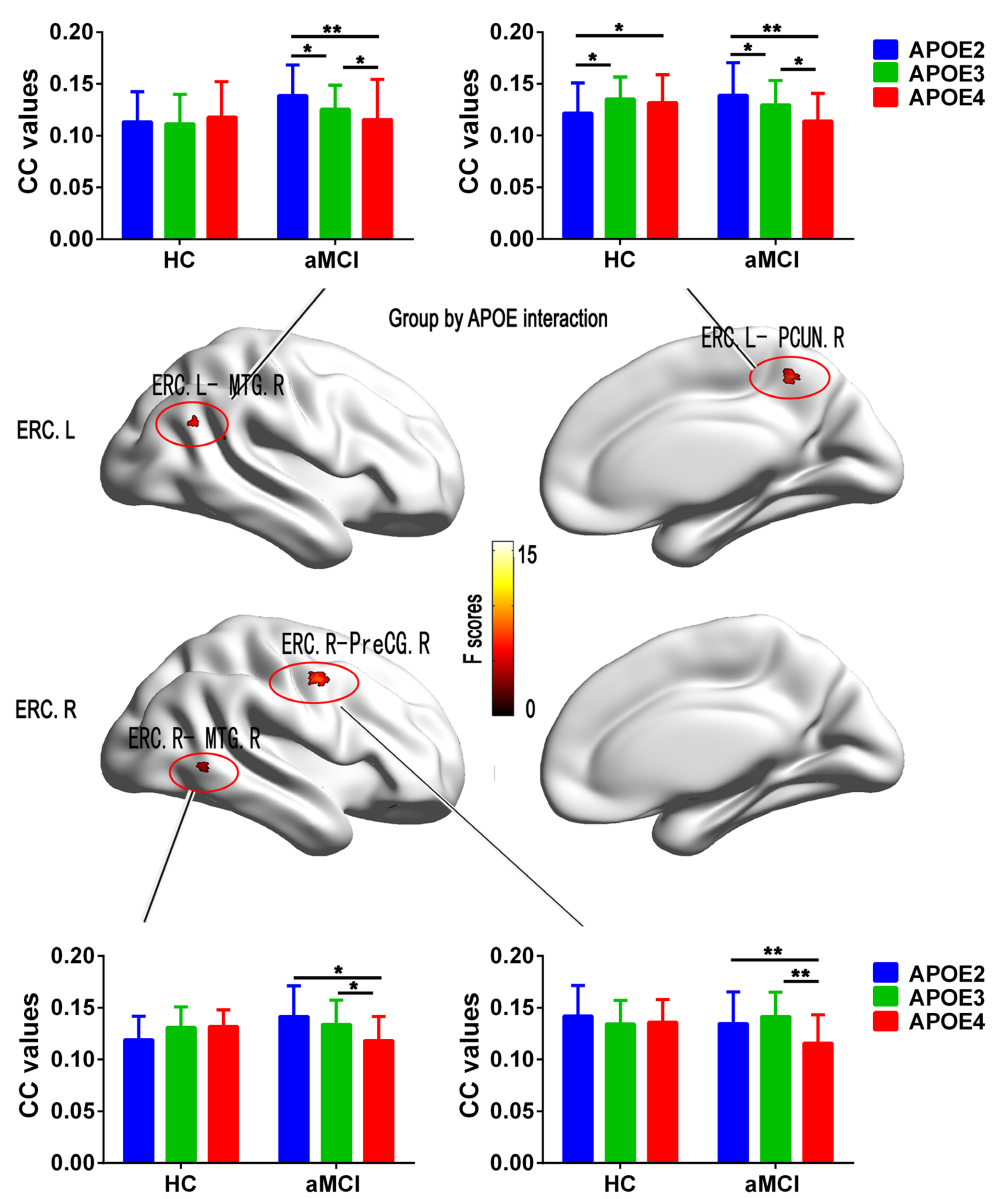
Results from the analysis of interaction for Group by APOE on functional connectivity associated with the ERC seed in Statistical Parametric Maps (SPM) (*p*< 0.05, FDR corrected) Histograms showing the mean values of functional connectivity extracted from the interaction brain regions respectively for APOE2 (blue), APOE3 (green), and APOE4 (red) in the aMCI patients and HC subjects. Bars are standard errors of the mean. Abbreviation: HC, healthy control; aMCI, amnestic mild cognitive impairment; ERC, entorhinal cortex; MTG, middle temporal gyrus; PCUN, precuneus; PreCG, precentral gyrus; CC, correlation coefficient; L, left, R, right.

The interaction of “group” × “APOE” was observed in the FC between left ERC and the right middle temporal gyrus (MTG), between left ERC and the right precuneus (PCUN), between right ERC and the right MTG, and between right ERC and the right precentral gyrus (PreCG) (Figure 2). Post hoc comparisons further showed that, in aMCI group, APOE2 showed higher FC in bilateral MTG, right PCUN and right PreCG than APOE3 and APOE4 (except similar FC with the right PreCG), and APOE4 showed lower FC in above-mentioned regions than APOE3 (Figure 2). But in the HC group, APOE2 showed lower FC in right PCUN than APOE3 and APOE4 (Figure 2). Moreover, in the ERC network, aMCI carried APOE4 showed decreased FC with the bilateral MTG and the right PCUN, and the right PreCG while APOE2 showed increased FC (except decreased FC with the right PreCG) compared to HC (*p* < 0.05, Figure 2). However, no any differences were observed in the above brain regions for APOE3 (*p* > 0.05). The results of the APOE genotypes comparisons in each group were provided in the complementary Figure S1.

### Functional connectivity patterns of the ERC network in the APOE ε4, ε3/ε3 and ε2

**Figure 3:**
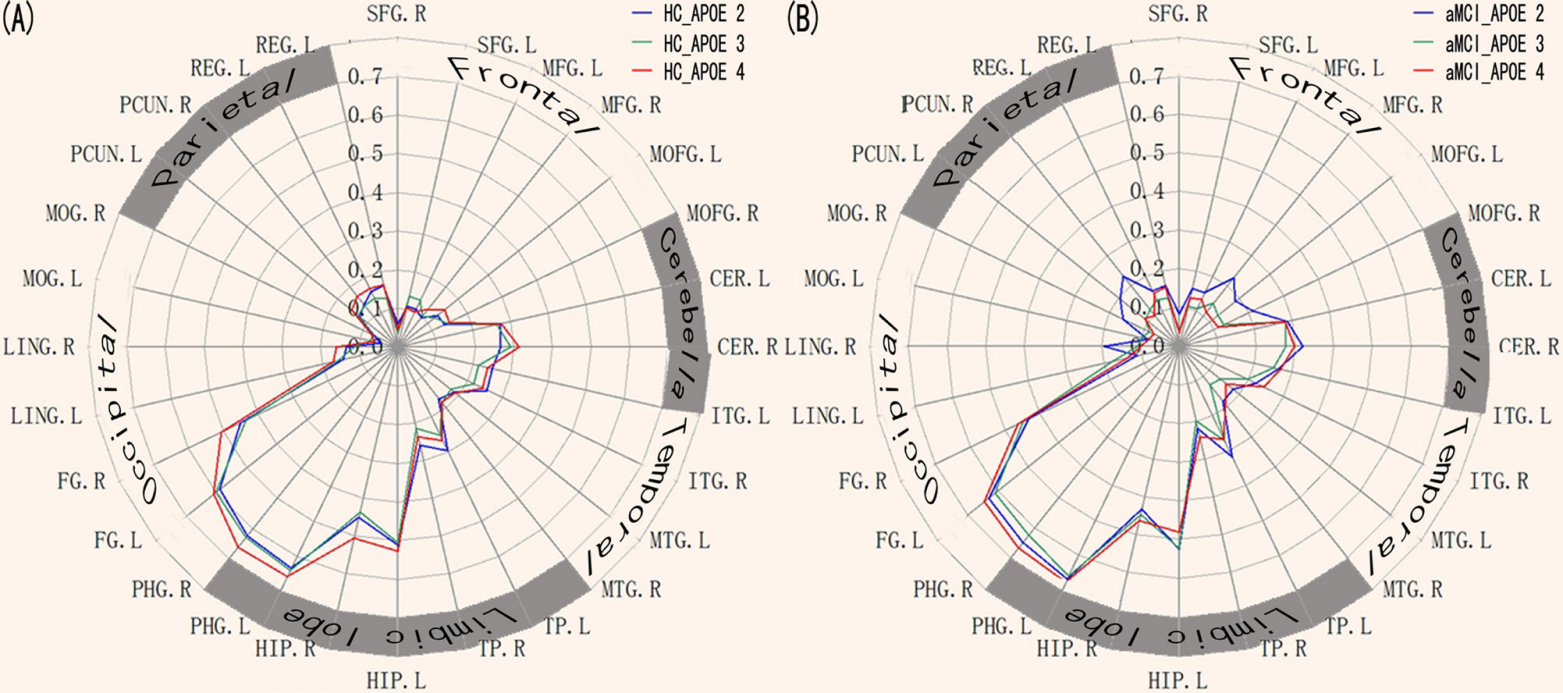
Heterogeneous functional connectivity associated with the ERC seed in APOE2, APOE3, and APOE4, respectively **A.** Schematic polar plot depicts different connectivity patterns of ERC seed with target ROIs distributed across the whole brain for APOE2, APOE3, and APOE4 in HC subjects. The concentric circles depict parameter estimates representing the connectivity strength. **B.** Schematic polar plots depict different connectivity patterns of ERC seed with target ROIs distributed across the whole brain for APOE2, APOE3, and APOE4, respectively, in the aMCI patients. Note that the data of functional connectivity are extracted from the only significant within-group connectivity regions for one or both groups. Abbreviations: ERC, entorhinal cortex; SFG, superior frontal gyrus; MFG, medial frontal gyrus; MOFG, medial orbital frontal gyrus; *CER, cerebellum;* ITG, inferior temporal gyrus; MTG, Middle temporal gyrus; TP, temporal pole; HIP, hippocampus; PHG, parahippocampal gyrus; FG, fusiform gyrus; LING, lingual gyrus; PCUN, precuneus; REG, rectus gyrus; MOG, Middle occipital gyrus; ROI, interest of region.

Schematic polar plots demonstrated that there were similar FC patterns among APOE genotypes by visual inspection (Figure 3A). The MANCOVA analysis demonstrated that the ERC network showed significantly strong connectivity with widely distributed brain regions in cerebella-temporal-limbic-occipital-parietal system (*p* < 0.05, FDR corrected). Interestingly, the post-hoc t-tests demonstrated that aMCI showed the differentially impaired connectivity patterns in the APOE 4, APOE3 and APOE2 (*p* < 0.05, Figure 3B, 3C, 3D).

**Figure 4:**
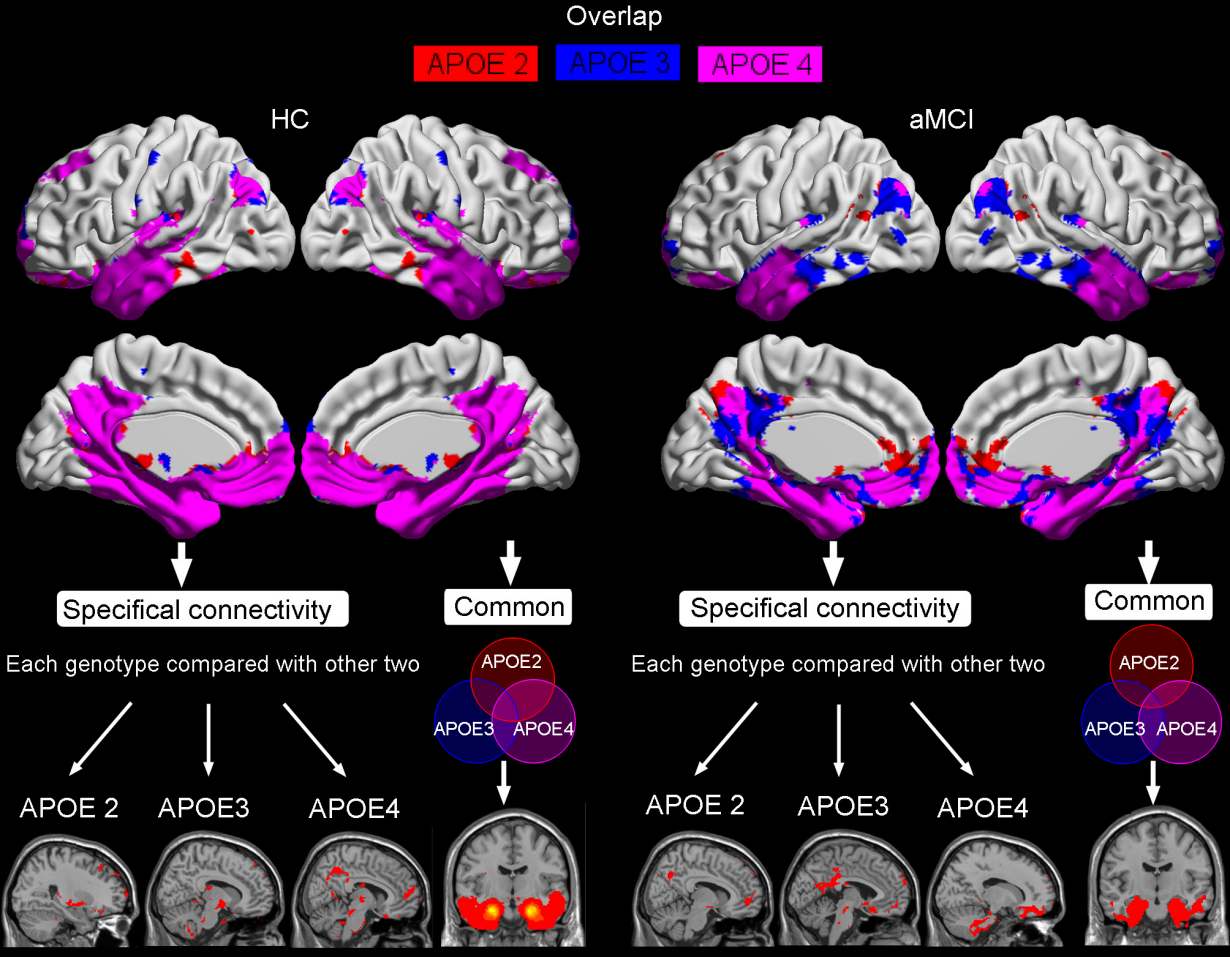
The overlap of resting-state intrinsic connectivity network maps in HC and aMCI produced by seeding ERC region, as well as the specifical and common connectivities of each APOE genotype compared with other two Shown are statistical*p*maps after correction for multiple comparisons (*p*< 0.05, FWE corrected). Abbreviations: ERC, entorhinal cortex; HC, healthy controls; aMCI, amnestic mild cognitive impairment.

These FC maps derived from the ERC seed in aMCI and HC were superimposed to illustrate their overlap and specificity between APOE genotypes (Figure 4). All three maps mainly overlapped in limbic lobe (parahippocampal gyrus), temporal lobe (middle temporal gyrus, superior temporal gyrus, inferior temporal gyrus), and fusiform. Compared with APOE3 and APOE4, APOE2 mainly showed most strong connectivity with frontal lobe (superior frontal gyrus and middle frontal gyrus), and middle temporal gyrus. APOE3 mainly showed most strong connectivity with postcentral gyrus, paracentral lobule, inferior parietal lobule, thalamus, insula, and cerebellum anterior lobe. APOE4 mainly showed most strong connectivity with precuneus, middle occipital gyrus, angular gyrus, superior parietal lobule, posterior cingulate, and medial orbitofrontal gyrus (Figure 4).

### Behavioral significance of the altered FC of ERC network on the interaction of APOE with aMCI

**Figure 5:**
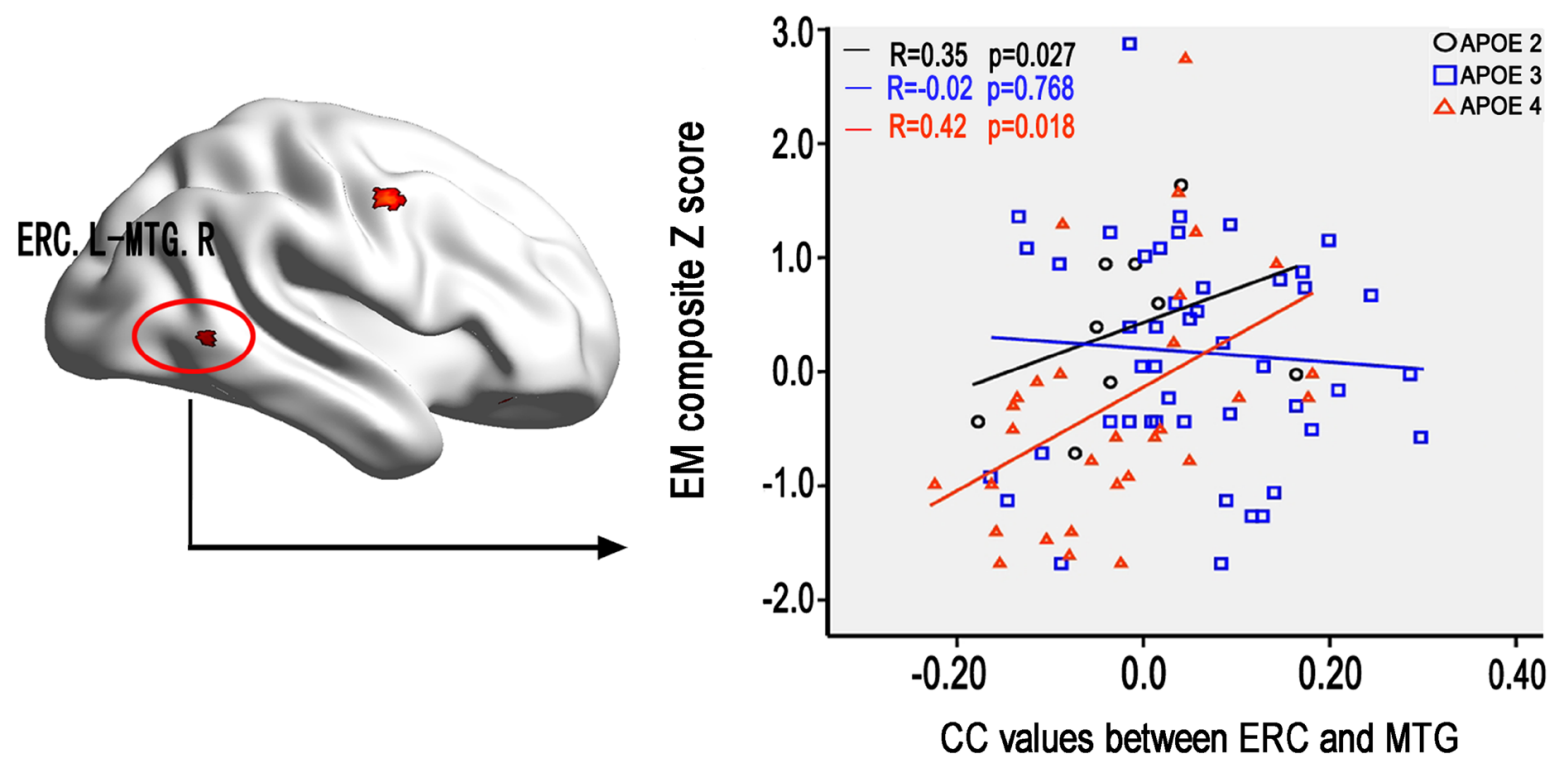
The effect of*APOE*genotype on the association between the altered ERC connectivity in aMCI and cognitive performance Scattergrams represent the correlations between cognition and resting-state functional connectivity of ERC in aMCI patients. Correlation coefficients (R) and p values are reported in each correlation graph. Abbreviations: ERC, entorhinal cortex; CC, correlation coefficient; EM, episodic memory; MTG, middle temporal gyrus; L, left; R, right; HC, healthy controls; aMCI, amnestic mild cognitive impairment.

The multivariate linear regression analysis demonstrated that the altered FC between left ERC and right MTG closely associated with the impairment of episodic memory in aMCI carried APOE ε4 and ε2 allele but not ε3/ε3 allele (Figure 5). However, no significant correlations were evident with respect to other cognitive function in aMCI. Additionally, no significant correlations were found between the FC in the same brain regions with *cognitive performance in HC subjects.*

## DISCUSSION

This study was to investigate the effects of APOEε2 and ε4 alleles on FC of ERC network and cognition in aMCI and to delineate the intrinsic FC patterns of the ERC with large-scale cortical networks. the interaction of “aMCI” × “APOE” was observed in the FC between left ERC and the right MTG, between left ERC and the right PCUN, between right ERC and the right MTG, and between right ERC and the right PreCG. Secondly, aMCI showed the differentially altered FC patterns of the ERC network among the APOE2, APOE3, and APOE4 compared to HC. Finally, the altered FC between left ERC and right MTG closely correlated with the impairment of episodic memory in aMCI with APOE4 and APOE2 but not APOE3. The study further indicated a significantly different impact of the APOE genotypes on altered FC of ERC network in specific brain regions associated with episodic memory in aMCI and provided new insights to understand the pathophysiology underlying the impairment of episodic memory in aMCI.

The findings further showed that aMCI with APOE4 showed reduced FC with the bilateral MTG and the right PCUN, and the right PreCG in the ERC network compared to HC. Evidence from animal and human studies has consistently identified that the ERC, known as the gateway to the medial temporal lobe, relays sensory information from the outer cortex of the brain to the hippocampus [8, 31]. It has been established that two neuropathways are needed to complete declarative memory formation: occipito-temporal visual object processing pathway (the “what” stream) [8]and parieto-temporal visuospatial pathway (the “where” stream) [33]. Our findings suggest that both visual object processing and visuospatial pathways exhibit the dysfunction of information processing for the formation of episodic memory. In addition, these findings were also consistent with the evidences from neuroimaging studies on the effects of APOEε4 allele, which have indicated that APOEε4 allele influences brain FC in clinically early phases of AD [22]. Furthermore, numerous studies have also consistently reported that APOEε4 can accelerate the rate of decrease of brain volumes [34], increased amyloid-β deposition [13, 35],increased numbers of neurofibrillary tangles [35], reduced GM volume [21], and the conversion from aMCI to AD [17]. Based on above-mentioned studies, as well as our findings, it suggests that APOEε4 allele may accelerate disease progression in specific brain regions of ERC network associated with episodic memory in aMCI.

Conversely, in aMCI patients, the APOEε2 carriers had increased FC with the bilateral MTG and the right PCUN in the ERC network (except decreased FC with the right PreCG) compared to HC subjects. Several studies have reported that the MTG-ERC and PCUN -ERC pathways play a key role in the visual object processing and visuospatial processing, respectively [8, 31, 33, 36]. Our findings may suggest that both visual object processing and visuospatial pathways exhibit information processing in an abnormal manner although the two pathways showed increased connectivity [8, 31, 33, 36]. In addition, numerous studies have widely indicated a protective clinical and pathological effect of APOEε2, such as slower functional decline in aMCI [21], improvement of episodic memory over time [20], fewer amyloid plaques [18, 35], fewer neurofibrillary tangles [37]. Based on above-mentioned studies, as well as our findings, it suggests a possible protective effect of the ε2 allele on the ERC network [38]. Furthermore, the neuroimaging evidence has reported an increased FC of ERC, which may suggest a compensatory mechanism in aMCI [27]. Therefore, converging evidence suggests that ε2-carriers might attempt to compensate for worsening neuropathological changes in aMCI, which may represent an attempted compensatory response to AD neuropathology. It further suggests that only APOEε2 allele plays a pivotal role in the compensatory mechanism locating at the right ERC- MTG -PCUN neural circuit in aMCI, but not APOEε4 allele. However, in this study we have not performed object perception test. Future study should be needed to identify this findings in the present study.

Interestingly, the present study showed that different effects of APOE on FC of ERC between HC and aMCI. That is, different effects of APOE genotypes could be found in aMCI, but in HC. This may suggest that each APOE allele plays different roles in FC of ERC under AD pathology, but normal aging did not find the effects of APOE allele. It is reason why APOE allele may interacted with AD pathology whit the progression of early AD. Then these effects of APOE allele were induced under AD pathology [14, 18, 35]. Furthermore, we found APOE the protective APOE ε2 allele appears to be against the disease [14, 15, 18, 19], whereas APOE ε4 allele accelerated the progression of early AD [13-15].

Given strong evidence for the opposite effects on intrinsic FC patterns of the ERC with large-scale cortical networks in aMCI, our findings raise questions as to the mechanism by which APOEε2 and APOEε4 are affecting neuropathology of aMCI. APOEε2 could have several different mechanisms that contribute to its protective effects. Firstly, numerous studies have identified a link between various isoforms of APOE and degradation of β-amyloid [14, 18, 35], which suggest that APOEε2 is significantly more effective at clearing soluble β-amyloid compared to APOEε4. This effective β-amyloid clearance mediated by APOEε2 may contribute to lower levels of oligomers or other toxic assembly states of β-amyloid [14]. Secondly, APOEε2 may directly interact with tau protein, the main constituent of neurofibrillary tangles and interact with tau to a different extent than the other APOE alleles [39]. The APOEε2 has a direct effect against neuritic plaques and an indirect consequence of its protective effect on the formation and spreading of neurofibrillary tangles in the neocortex [14], which implies a synergistic interaction between tangles and plaques [14], and determine a lower Braak stage by protecting against the accumulation of soluble Aβ oligomeric species and plaques [14, 40] and by protecting against synaptotoxicity and neurotoxicity of existing plaques downstream Aβ accumulation [40]. By contrast, APOEε4 may increase the amount of synaptotoxic soluble Aβ oligomeric species[14, 40] and impair Aβ clearance [14, 40]. It has been well reported that APOEε4 may be considered to play a key role as an initiator of the amyloid cascade that ultimately leads to fully established AD [41].

Associations between disconnect of ERC and right MTG and neuropsychological performance were identified to support the relationship between intrinsic FC and cognition in aMCI with ε4 and ε2 allele. In addition, we have not found association between FC and episodic memory. It is reason why aMCI with APOE3 showed no alerted FC than HC subjects. Therefore, FC in aMCI with APOE3 was not influenced by the AD pathology. These findings further showed that disconnect of left ERC and right MTG positively associated with the impairment of episodic memory but not other any cognitive functions in aMCI with ε4 and ε2 allele, which suggests that the altered ERC-MTG pathway is associated with the dysfunction of the formation of episodic memory [29]. Furthermore, a recent neuroimaging study has identified a direct influence of resting-state FC on the association between Aβ-pathology and cognitive impairment [42]. Numerous studies have consistently revealed a link between various isoforms of APOE and degradation of β-amyloid [14, 18, 35]. Therefore, we propose that both *APOE*ε2 and *APOE*ε4 have an independent impact on cognition and each indirectly affects cognition through its protective or toxic effects on brain intrinsic FC changes [14]. Converging evidence suggests that APOE may indirectly modulate the ERC-MTG neural pathway of episodic memory in aMCI patients. However, care should be taken to explain the association between functional connectivity and episodic memory in aMCI with APOE2 carriers. Because there was a small sample of APOE2 carriers (i.e. 9 APOE2 subjects).

There are limitations in the current study. First, the aMCI sample size was relatively small in ε2-carriers, which might have affected the results. Second, the present study was a cross-sectional study. The potential effects of the ε4 genotype may be a dynamic phenomenon and more impaired the FC deficits described may well refer to the state of the disease rather than certain subtypes, so additional longitudinal studies will be very helpful in determining whether the potential effects of the ε4 in aMCI is specifically associated with a more rapid course of AD. Finally, the present study merged hetero-and homozygotes into one group not to assess the dose-dependent effect of APOE ε2 and ε4 alleles. Future study was need to assess the dose-dependent effect of APOE alleles.

In conclusion, this study provides the first evidence that the effects of*APOE*on the ERC network are closely linked to the role of this gene on AD risk, which aMCI with ε4-carriers can accelerate the early pathological progression of network-based mechanisms of late-life cognitive decline while ε2-carriers may play a protective role in contributing to a compensatory mechanism. Furthermore, APOEε2 and APOEε4 have an independent impact on cognition and each indirectly affects cognition through its protective or toxic effects on the ERC network. It further suggests that APOE can appear to directly affect the ERC-MTG neural pathway associated with the impairment of episodic memory, which can be an important functional imaging indicator for early stage changes and progression of aMCI.

## MATERIALS AND METHODS

### Subjects

This study recruited 181 elderly individuals, including 85 aMCI-multiple domain subjects and 96 HC subjects. Two aMCI and eight HC subjects were excluded due to excessive movement [number of instantaneous movements [43, 44] > 0.5 mm exceeded the mean plus 1 standard deviation (SD)]. Finally, the remaining 88 HC subjects include 15 APOE ε2ε3 (abbreviated as APOE2), 40 APOE ε3 homozygotes (abbreviated as APOE3) and 33 APOE ε3ε4 (abbreviated as APOE4), and 83 aMCI include 9 APOE2, 44 APOE3 and 30 APOE4 (23 APOE ε3ε4 genotype and 7 APOE ε4 homozygote). In this study, due to the inclusion and exclusion criteria used to choose subjects, matched conditions between the groups, and the excessive movement, the distribution of the APOE subtypes was different from that of the general population, however, the distribution of the APOE subtypes in our total samples was consistent with that of the general population. Written informed consent was obtained from all of the participants, and the study was approved by the responsible Human Participants Ethics Committee of the Affiliated ZhongDa Hospital, Southeast University.

All aMCI-multiple domain subjects met the diagnostic criteria proposed by Petersen and colleagues [45] and the revised consensus criteria of the International Working Group on aMCI [46], including (1) subjective memory impairment corroborated by the subject and an informant, (2) objective memory performance documented according to an Auditory Verbal Memory Test-delayed recall score that was within ≤ 1.5 SD of age- and education-adjusted norms (the cutoff was ≤ 4 correct responses on 12 items for ≥ 8 years of education), (3) in addition to memory dysfunction, objective evidence of cognitive impairment in one or more of the following functions: language, execution, attention, or other cognitive domains [47], (4) normal general cognitive function as evaluated by a Mini mental state exam (MMSE) score of 24 or higher, (5) a Clinical Dementia Rating of 0.5, with at least a 0.5 in the memory domain, (6) no or minimal impairment in daily living activities, and (6) absence of dementia, symptoms that were not sufficient to meet the criteria of the National Institute of Neurological and Communicative Disorders and Stroke or the AD and Related Disorders Association criteria for AD. Exclusion criteria were as follows: (1) a past history of known stroke (modified Hachinski score of > 4), alcoholism, head injury, Parkinson's disease, epilepsy, major depression (excluded by Self-Rating Depression Scale), or other neurological or psychiatric illness (excluded by clinical assessment and case history), (2) major medical illness (e.g., cancer, anemia, and thyroid dysfunction), (3) severe visual or hearing loss, and (4) T2-weighted MRI showing major white matter (WM) changes, infarction, or other lesions (two experienced radiologists analyzed the scans).

The HC subjects were required to have a clinical dementia rating of 0, an MMSE score of ≥ 26, and a delayed recall score of > 4 for those with ≥ 8 years of education. These participants were matched by subject to aMCI subjects. All subjects underwent a standardized clinical interview including demographic inventory, medical history and neurological and mental status examination. Group-specific demographics and neuropsychological characteristics are provided in Table 1.

### Neuropsychological assessments

As our previously published described [24, 36], all subjects underwent a standardized clinical interview and comprehensive neuropsychological assessments performed by neuropsychologists (Dr. Shu, Wang, and Liu). These tests were used to evaluate general cognitive function, episodic memory, information processing speed, executive function, and visuo-spatial function, respectively. The details regarding these assessments are provided in SI Methods.

### APOE genotyping

As our previously published described [21], genomic DNA of each subject was extracted from 250ul EDTA-anticoagulated blood using a DNA direct kit (Tiangen, China). A polymerase chain reaction-based restriction fragment length polymorphism (PCR-RFLP) assay was applied to detect the allele of rs7412 and rs429358, respectively, the haplotype of which determined the APOE genotype ultimately. The details of the specific process are provided in SI Methods.

### MRI data acquisition

MRI images were acquired using a 3.0 Tesla Trio Siemens scanner (Siemens, Erlangen, Germany) with a 12-channel head-coil at ZhongDa Hospital Affiliated to Southeast University. The details regarding image acquisition parameters are provided in SI Methods.

### Image preprocessing

Data analyses of groups were performed using SPM8 (available at: http://www.fil.ion.ucl.ac.uk/spm). Briefly, this preprocessing included removal of the first ten volumes, slice timing correction, and head motion correction [43, 44]. Participants with excessive head motion (cumulative translation or rotation of more than 2.0 mm or 2.0° and mean point-to-point translation or rotation of more than 0.5 mm or 0.1°) were excluded [43, 44, 48-50]. To spatially normalize the rs-fcMRI data, the T1-weighted images were used to register the functional data to their corresponding anatomical image, and the resulting aligned T1 dataset was transformed into Montreal Neurological Institute (MNI) space. To improve the coregistration of the rs-fcMRI data, a custom T1 template was built by averaging the normalized anatomical images across all subjects. Finally, the normalized functional images were created by applying the transformation of the T1 images to the customized T1 template. Notably, such a custom template-based registration procedure could reduce the inaccuracy of the spatial normalization of functional volumes due to GM atrophy in aMCI and HC. Functional images were resampled to 2×2×2 mm^3^ voxels and spatially smoothed using a 4-mm full-width half-maximum (FWHM) Gaussian kernel. Linear detrending and temporal band-pass filtering (0.01-0.08 Hz) were applied to reduce the effect of low-frequency drifts and high-frequency physiological noise. Finally, several nuisance variables, including six head motion parameters, global mean signal [51], cerebrospinal fluid, and white matter (WM) signal were removed by multiple linear regression analysis. No significant differences between groups were observed in quality assurance (QA) parameters (*p* > 0.05). The details regarding QA, including assessment of susceptibility, GM loss effect, and head motion effects, are provided in SI Methods.

### Functional connectivity analyses

The precise location of the ERC [MNI space: ±26, -16, -28] was selected to generated a four-millimeter radius spherical seed region and determined by convergent evidence from previous studies in animals and humans [33, 52]. Individual averaged time courses for all voxels within ERC region were extracted based on the coregistered seed region as the reference time course, and voxelwise cross-correlation analysis was then carried out between the averaged time courses of all voxels within the seed region and the whole brain within the GM mask. The GM mask was created by thresholding (a probability threshold of 0.2) the GM probability map obtained from all subjects in this study [53]. A Fisher's z-transform was then applied to improve the normality of the correlation coefficients. For each subject, we obtained two z-score maps that represented the intrinsic FC patterns of ERC.

### Statistical analyses

#### Demographic and neuropsychological data

The statistical analyses were performed using SPSS 17.0 software (SPSS Inc., Chicago, IL, USA). The multivariate analysis of covariance (MANCOVA, group × APOE) and chi-square test were used to test the differences of demographic data and neuropsychological performance between aMCI and HC (*p* < 0.05). And post-hoc t-tests with Bonferroni correction were used to further investigate differences in each APOE subgroup (*p* < 0.05).

#### Group-level intrinsic connectivity analysis

To determine the patterns of FC of ERC seed, the spatial maps of FC in each group were submitted to a random-effect analysis using one-sample t-tests with age, gender, and years of education treated as covariates at a stringent threshold of *p* < 0.001 using family-wise error (FWE) correction at the whole-brain to reveal regions most robustly correlated with ERC seed.

In addition, differences in voxel-wise group comparisons of FC were tested using a 2 × 3 MANCOVA with age, gender, years of education, and voxel-wise GM volumes treated as covariates at a p < 0.05 corrected by false discovery rate (FDR). And post-hoc t-tests were used to further investigate differences of APOE alleles within each group as a significant interaction of group by APOE. A statistical threshold of p (uncorrected) < 0.005 and cluster extent k > 100 voxels (800 mm3) was used to achieve a corrected statistical significance of *p* < 0.05, determined by Monte-Carlo simulation (see program AlphaSim by D. Ward).

To examine the potential overlap between the connectivity maps of different APOE genotypes derived from the ERC seed, the three SPM-T maps from the one sample *t*-tests were thresholded as indicated above, binarized, subtracted and summed, resulting in the so-called “specifical” or “overlapping” regions.

To characterize FC patterns of ERC seed, schematic polar plots were then used to summarize overall FC patterns of APOE genotypes with target regions throughout the whole-brain. A 2×3 MANCOVA with age, gender, years of education, and voxel-wise GM volumes treated as covariates was used to test the difference of FC patterns of ERC seed for APOE genotypes between aMCI and HC (*p* < 0.05, FDR corrected). A post-hoc Student's t-test with FDR correction for each two pairs of groups was performed to further investigate differences in any statistical significance for MANCOVA (*p* < 0.05).

#### The effect of APOE genotype on the association between the altered ERC connectivity and cognition

The averaged FC strengths of the regions showing altered ERC connectivity in aMCI compared to HC were extracted from -score maps. The multiple linear regression model with stepwise method was used to examine the effect of APOE genotype on the association between the altered ERC connectivity in aMCI compared with HC and cognition. To increase the statistical power by reducing random variability, this study composited the neuropsychological tests into 4 cognitive domains and transformed the raw scores into 4 composite Z scores. Bonferroni correction for multiple comparisons was performed (*p* < 0.05). Details on the composites of neuropsychological tests and the transformation of raw scores are provided in SI Methods.

## SUPPLEMENTARY INFORMATION FIGURE


