# Supplementary material for: Protective effect of APOE epsilon 2 on intrinsic functional connectivity of the entorhinal cortex is associated with better episodic memory in elderly individuals with risk factors for Alzheimer's disease
Source: Oncotarget. 2016 Aug 14;7(37):58789–801. doi: 10.18632/oncotarget.11289 (PMC5312276; doi:10.18632/oncotarget.11289)
Supplement: Supplementary file 1 [file oncotarget-07-58789-s001.pdf]

Protective effect of APOE epsilon 2 on intrinsic functional connectivity of the entorhinal cortex is associated with better episodic memory in elderly individuals with risk factors for Alzheimer's disease

Supplementary Information

SI methods

Neuropsychological assessments

As our previously published described [1, 2], all subjects underwent a standardized clinical interview and comprehensive neuropsychological assessments performed by neuropsychologists (Dr. Shu, Wang, and Liu), including the Mini-Mental State Examination (MMSE), Mattis Dementia Rating Scale-2 (MDRS-2), Auditory Verbal Learning Test – immediate recall (AVLT-IR), Auditory Verbal Learning Test – 5-min delayed recall (AVLT-5-min-DR), Auditory Verbal Learning Test – 20-min delayed recall (AVLT-20-min-DR), Logical Memory Test-immediate recall (LMT-IR), Logical Memory Test – 20-min delayed recall (LMT-20-min-DR), Rey-Osterrieth Complex Figure Test (ROCFT), Rey-Osterrieth Complex Figure Test – 20-min delayed recall (ROCFT-20min-DR), Trail-Making Tests A and B (TMT-A and B), Digital Symbol Substitution Test (DSST), Digit Span Test (DST), Stroop Color and Word Test A, B, and C, Verbal Fluency Test (VFT), Semantic Similarity (Similarity) test, and Clock Drawing Test (CDT).

Process for Determining the APOE Genotype

As our previously published described [1, 3], a polymerase chain reaction-based restriction fragment length polymorphism (PCR-RFLP) assay was applied to detect the alleles of rs7412 and rs429358, respectively. The amplification reaction system contained 1 × GC buffer I (TAKARA), 2.0-mM Mg2+, 0.2-mM dNTP, 1-unit HotStarTaq polymerase (Qiagen Inc.), 1-μl genomic DNA and 1-μl (2-μM) primer for each allele (for rs429358, forward primer: AGGGCGCTGATGGACGAGAC, reverse primer: GCCCCGGCCTGGTACACT; for rs7412, forward primer: GGCGCGGACATGGAGGAC, reversed primer: GCCCCGGCCTGGTACACT). PCR cycling conditions were set as follows: 1) 95ºC lasted for 15 min; 2) we performed 11 cycles, each of which included a) 94ºC for 20s, b) we kept the temperature at 0.5 ºC below the melting temperature for 40s, c) 72ºC for 1min 40s; 3) we performed another 24 cycles, each of which included a) 94ºC for 20s, b) we kept the temperature at 6ºC below the melting temperature for 40s, c) 72ºC for 1.5min; 4) 72ºC for 2 min. Amplification was carried out on 2720 Thermal Cycler (ABI). Then, 10-μl amplified product was digested with 1-unit restriction endonuclease (AflⅢ for rs429358 and HaeⅡ for rs7412) at 37ºC overnight. Finally, the digested product was diluted tenfold and analyzed by capillary

electrophoresis to detect the alleles of rs429358 and rs7412. As a result, the APOE genotype was determined by the haplotype of rs429358 and rs7412. APOE ε2 allele was recognized by rs429358-T and rs7412-T, APOE ε3 allele was identified by rs429358-T and rs7412-C, and APOE ε4 allele was defined by rs429358-C and rs7412-C.

Image Acquisition

MRI images were acquired using a 3.0 Tesla Trio Siemens scanner (Siemens, Erlangen, Germany) with a 12-channel head-coil at Zhongda Hospital Affiliated to Southeast University. Resting-state functional images including 240 volumes were obtained using a gradient-recalled echo-planar imaging (GRE-EPI) sequence, with repetition time (TR) = 2000 ms, echo time (TE) = 25 ms, flip angle (FA) = 90°, acquisition matrix = 64 × 64, field of view (FOV) = 240 mm × 240 mm, thickness = 4.0 mm, gap = 0 mm, number of slices = 36, and voxel size = 3.75 × 3.75 × 4 mm^3. High-resolution T1-weighted axial images covering the whole brain were acquired by 3D magnetization prepared rapid gradient echo (MPRAGE) sequence as described below: TR = 1900 ms, TE = 2.48 ms; FA = 9°, acquisition matrix = 256 × 256, FOV = 250 × 250 mm, thickness = 1.0 mm, gap = 0 mm, number of slices = 176, and voxel size = 1 × 1 × 1 mm^3. Additionally, routine axial T2-weighted image were acquired to rule out subjects with major WM changes, cerebral infarction or other lesions using flair sequence as described below: TR = 8400 ms, TE = 94 ms, FA= 150°, acquisition matrix = 256×256, FOV = 230×230 mm, thickness = 5.0 mm, gap = 0 mm, and number of slices = 20.

Quality assurance (QA)

Assessment of susceptibility artifacts

Previous studies have indicated that brain areas in the MTL directly above the petrous bone especially tend to signal loss [4]. To assess the effects of susceptibility artifacts in our data, the signal-to-noise ratio (SNR) was computed for each voxel by averaging the signal intensity across all the target-atlas normalized BOLD runs and dividing it by the standard deviation (SD) over time [5]. To avoid variability in EPI timeseries cause of susceptibility artifact, the ERC seed was thresholded to exclude voxels with mean signal in the intensity-normalized EPI timeseries below 3000, corresponding approximately to a SNR of 20 [6]. Thresholds resulted in the rejection of no more than 5% of voxels in the ROI. Then the thresholded ROI was further used to perform FC analyses.

Gray matter loss effect

To avoid the interpretation on the differences of FC from the anatomical atrophies in the patients, voxelwise GM volumes were addressed as covariates in the further FC analysis. Voxel-based morphometry (VBM) analysis was performed using VBM8 toolbox in SPM8 (VBM8 toolbox, http://dbm.neuro.uni-jena.de/vbm). Briefly, the individual T1-weighted images were segmented into GM, WM and CSF, and then normalized to the MNI space. The normalized GM volume maps (modulated images) were resampled to the same grid as the functional image. Finally, the voxelwise GM values were regressed out as the nuisance regressor from the FC values to control the influence of GM volume on the FC strength. The voxewise GM volume correction was performed for each subject. Then, a two-sample t-test was performed to determine whether the GM was atrophied in aMCI, controlling for age, gender, and years of education.

Head motion effects

To minimize the influence of head motion both at the individual and at the group levels, three approaches were employed in QA measures. First, the head motion effects were regressed out, which were calculated as the root mean squared (rms) head displacement or rotation (in mm or °) derived from the motion-correction procedure [7]. Second, a ‘scrubbing’ procedure was carried out to scrub frames (volumes) with excessively high whole-brain rms signal change over time in the preprocessed rs-fcMRI data for each individual [8-10]. These frames were subsequently removed from rs-fcMRI analysis. The fraction of frames so removed was < 5 % in each group (no significant effect of group as factor on fraction of frames removed). Overall, 2 aMCI and 4 HC had a large proportion of high-noise frames (> 20% frames identified as contaminated) and were therefore excluded from the analysis. Third, additional QA measures included rms head displacement or rotation (in mm or °) and the voxel-wise time series SD averaged over the whole brain [11]. We referred to a prior study [12] to empirically determine the exclusion criteria for QA measure with the objective of achieving QA parameter distribution equivalence between groups while maximizing the number of included subjects. Overall, 4 HC subjects with a mean preprocessed rs-fcMRI signal 2.5% SD (after nuisance regression) or rms movement or rotation exceeding 2.0 mm or 2.0° and mean frame-to-frame rms movement or rotation more than 0.5 mm or 0.1° were also excluded. No significant differences between groups were observed in QA parameters ($p > 0.05$).

Statistical analysis

Group-level intrinsic connectivity analysis

A repeated-measure ANOVA with age, gender, years of education, and GM volumes treated as covariates was used to test the difference of FC patterns for APOE genotypes in HC subjects. The repeated-measure ANOVA have one between-subjects factor (APOE genotypes) and one within-subjects factor (target brain regions). A post-hoc Student's t-test with FDR correction for each two pairs between APOE genotypes was carried out to further investigate differences as any statistical significance for ANOVA. $p < 0.05$ *was required for statistical significance.*

The relationships between the altered ERC pattern and neuropsychological performance

To increase statistical power by reducing random variability, this study composited the neuropsychological tests into 4 cognitive domains and transformed the raw scores into 4 composite Z scores, as previously described [1, 2, 13]. First, for each neuropsychological test, the individual raw scores were transformed to Z scores, according to the mean and standard deviation of the scores for all subjects. Notably, for tests measured by timing, including TMT-A, TMT-B, Stroop A, Stroop B, and Stroop C, the raw scores were defined as the reciprocal of the time required for the test. Then, each cognitive domain's composite Z score was determined by averaging the Z scores related to the tests. We divided these tests into 4 cognitive domains: episodic memory (3 tests, including AVLT-20-min DR, LMT-20-min DR, and ROCFT-20-min DR), information processing speed (4 tests, including DSST, TMT-A, Stroop A, and Stroop B), visuospatial function (2 tests, including ROCFT and CDT), and executive function (5 tests, including VFT, DST-backward, TMT-B, Stroop C, and Similarity). All statistical procedures utilized SPSS 17.0 software (SPSS Inc., Chicago, IL, USA). Bonferroni correction for multiple comparisons was performed in the significance level considered at $p < 0.05$.

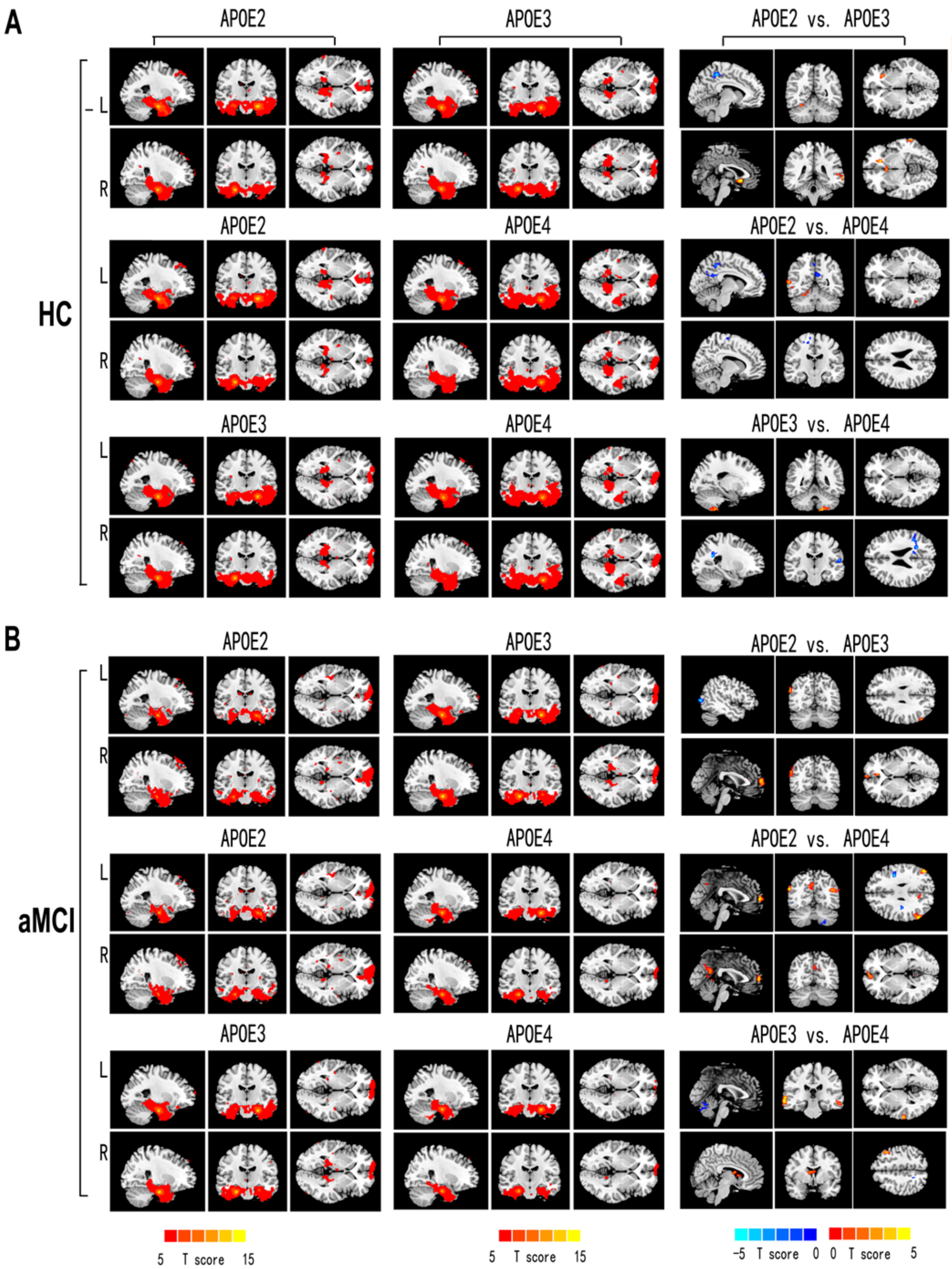

Figure S1: Resting-state functional connectivity patterns within APOE genotypes and between APOE genotypes maps in the entorhinal cortex network in HC (A) and aMCI (B). The first and second columns show the within-APOE genotypes statistical maps for APOE2 carriers, APOE3, and APOE4, respectively, with statistical threshold set at $p_{corrected} < 0.01$, corrected by FWE. Last column shows the between- APOE genotypes statistical maps, with statistical threshold set at $p_{corrected} < 0.05$, corrected by false discovery rate (FDR). Warm and blue colors indicate decreased and increased functional connectivity in aMCI subjects compared to HC subjects. Color bar is presented with T score.

References

1. Shu H, Shi Y, Chen G, Wang Z, Liu D, Yue C, Ward BD, Li W, Xu Z, Chen G, Guo Q, Xu J, Li SJ and Zhang Z. Opposite Neural Trajectories of Apolipoprotein E 4 and 2 Alleles with Aging Associated with Different Risks of Alzheimer's Disease. Cerebral cortex. 2016; 26(4):1421-1429.
2. Chen J, Duan X, Shu H, Wang Z, Long Z, Liu D, Liao W, Shi Y, Chen H and Zhang Z. Differential contributions of subregions of medial temporal lobe to memory system in amnestic mild cognitive impairment: insights from fMRI study. Scientific reports. 2016; 6:26148.
3. Chen J, Shu H, Wang Z, Liu D, Shi Y, Zhang X and Zhang Z. The interaction of APOE genotype by age in amnestic mild cognitive impairment: a voxel-based morphometric study. Journal of Alzheimer's disease. 2015; 43(2):657-668.
4. Kahn I, Andrews-Hanna JR, Vincent JL, Snyder AZ and Buckner RL. Distinct cortical anatomy linked to subregions of the medial temporal lobe revealed by intrinsic functional connectivity. Journal of neurophysiology. 2008; 100(1):129-139.
5. Murphy K, Bodurka J and Bandettini PA. How long to scan? The relationship between fMRI temporal signal to noise ratio and necessary scan duration. NeuroImage. 2007; 34(2):565-574.
6. Libby LA, Ekstrom AD, Ragland JD and Ranganath C. Differential connectivity of perirhinal and parahippocampal cortices within human hippocampal subregions revealed by high-resolution functional imaging. The Journal of neuroscience. 2012; 32(19):6550-6560.
7. Zuo XN, Kelly C, Di Martino A, Mennes M, Margulies DS, Bangaru S, Grzadzinski R, Evans AC, Zang YF, Castellanos FX and Milham MP. Growing together and growing apart: regional and sex differences in the lifespan developmental trajectories of functional homotopy. The Journal of neuroscience. 2010; 30(45):15034-15043.
8. Sheline YI, Raichle ME, Snyder AZ, Morris JC, Head D, Wang S and Mintun MA. Amyloid plaques disrupt resting state default mode network connectivity in cognitively normal elderly. Biological psychiatry. 2010; 67(6):584-587.
9. Power JD, Barnes KA, Snyder AZ, Schlaggar BL and Petersen SE. Spurious but systematic correlations in functional connectivity MRI networks arise from subject motion. NeuroImage. 2012; 59(3):2142-2154.
10. Power JD, Schlaggar BL and Petersen SE. Recent progress and outstanding issues in motion correction in resting state fMRI. NeuroImage. 2015; 105:536-551.
11. de Calignon A, Polydoro M, Suarez-Calvet M, William C, Adamowicz DH, Kopeikina KJ, Pitstick R, Sahara N, Ashe KH, Carlson GA, Spires-Jones TL and Hyman BT. Propagation of tau pathology in a model of early Alzheimer's disease. Neuron. 2012; 73(4):685-697.
12. Brier MR, Thomas JB, Snyder AZ, Benzinger TL, Zhang D, Raichle ME, Holtzman DM, Morris JC and Ances BM. Loss of intranetwork and internetwork resting state functional connections with Alzheimer's disease progression. The Journal of neuroscience. 2012; 32(26):8890-8899.
13. Xie C, Bai F, Yu H, Shi Y, Yuan Y, Chen G, Li W, Chen G, Zhang Z and Li SJ. Abnormal insula functional network is associated with episodic memory decline in amnestic mild cognitive impairment. NeuroImage. 2012; 63(1):320-327.
